# Avoid the Peel: Citrus Fruit Bezoar Causing Intestinal Perforation

**DOI:** 10.7759/cureus.64262

**Published:** 2024-07-10

**Authors:** Wayne Tse, William Hope, Ryan Johnson, Jeannie Rivers, Thomas Miller

**Affiliations:** 1 Surgery, Central Virginia Veterans Affairs (VA) Health Care System, Richmond, USA; 2 Surgery, Virginia Commonwealth University School of Medicine, Richmond, USA; 3 Surgery, Lancaster General Hospital, Lancaster, USA; 4 Surgery, Loyola University Medical Center, Chicago, USA

**Keywords:** kumquats, bezoar, citrus fruit, intestinal perforation, intestinal obstruction

## Abstract

We report a highly unusual case of small bowel obstruction in an 86-year-old man from ingestion of a citrus fruit, known as kumquats, which led to intestinal perforation and peritonitis. He initially presented with a one-day history of diffuse abdominal pain associated with nausea and feculent emesis after eating whole pieces of unpeeled kumquats. When symptoms of peritonitis evolved with a blood lactate of 5.1 mg/dL, he was urgently taken to the operating room for exploration. Multiple areas with fibrous exudates and full-thickness ulceration were encountered along the distal jejunum and proximal ileum, with a partially obstructing intraluminal mass in the distal ileum. Treatment involved resection of 70 cm of non-viable bowel, removal of the intraluminal mass, and surgical re-establishment of intestinal continuity. Unpeeled kumquats were confirmed to have caused these intestinal findings. The patient did well following the operation and has had no further problems referred to by this management.

## Introduction

Ingestion of food is generally an effortless process that proceeds smoothly and may be very pleasurable, depending on how tasty the food is. After being swallowed, the contents eaten move down the esophagus, enter the stomach, and then the small intestine, where these organs participate in their breakdown into smaller components to initiate effective digestion and absorption so that nutrition and energy are provided to the body [1,2]. Although bowel obstruction can disrupt this process, it is distinctly uncommon unless an individual has undergone a previous abdominal operation in which adhesions have formed, or abdominal wall hernias exist through which portions of the intestine can incarcerate [3,4], resulting in small bowel obstruction.

We present a case of a patient who seemingly had uneventfully eaten a citrus fruit where normal digestion went badly wrong, resulting in an emergency intestinal operation.

## Case presentation

An 86-year-old man with a history of hypertension, diabetes, hyperlipidemia, aortic stenosis, and prostate cancer (treated some 19 years previously with radiation therapy) presented to the emergency department at our Veterans Hospital with a one-day history of diffuse abdominal pain associated with nausea and feculent emesis. He had no prior abdominal surgery, abdominal wall hernias, or family history of colorectal cancer.

**Figure 1:**
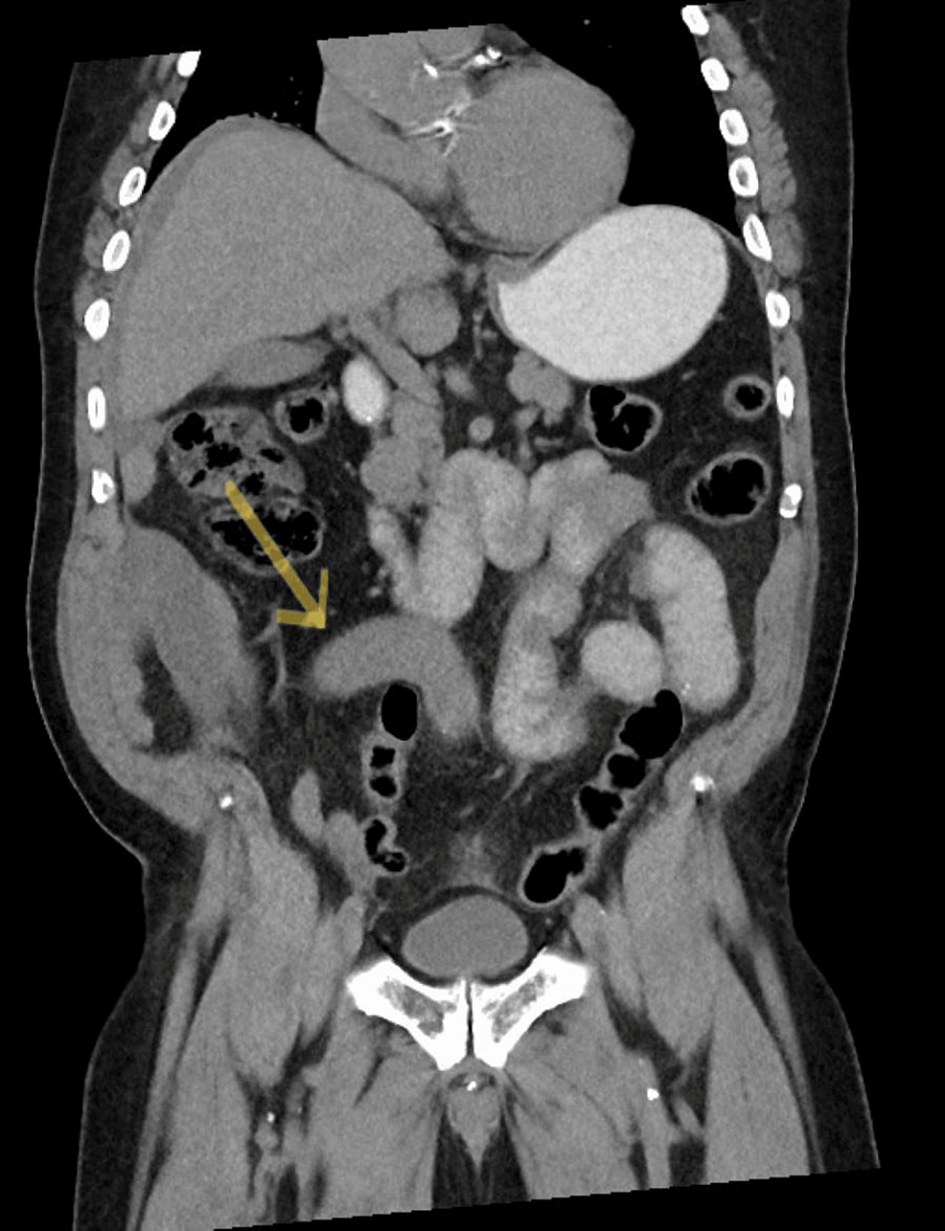
Coronal CT abdomen/pelvis with oral contrast showed mild small bowel dilation with a transition point (see arrow) lateral and anterior to the right paracolic gutter. No distal oral contrast is noted.

**Figure 2:**
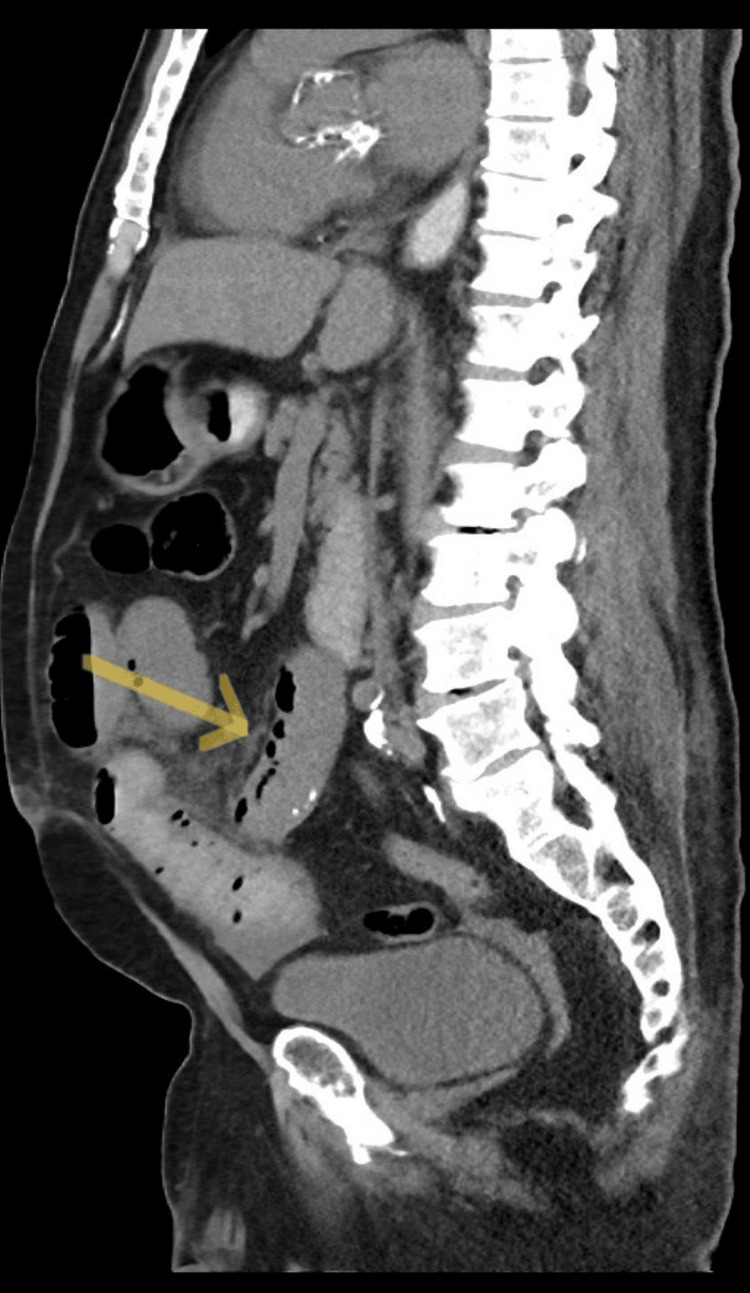
CT scan shows evidence of pneumatosis (see arrow) in portions of bowel ultimately resected.

The patient was afebrile and normotensive but ill, appearing on the initial evaluation. His abdomen was distended with right upper quadrant tenderness and rebound, voluntary guarding, and signs of peritonitis. Laboratory studies showed no leukocytosis but an elevated creatinine of 2.2 mg/dL (baseline of 1.3 mg/dL) and a lactate of 5.1 mg/dL (normal is 0.4-2.0). A CT scan of the abdomen and pelvis with PO contrast was obtained (Figure 1 and Figure 2). This scan showed mild small bowel dilation with a transition point lateral and anterior to the right paracolic gutter, indicating intestinal obstruction. Some portions of the intestine in this area also showed pneumatosis.

**Figure 3:**
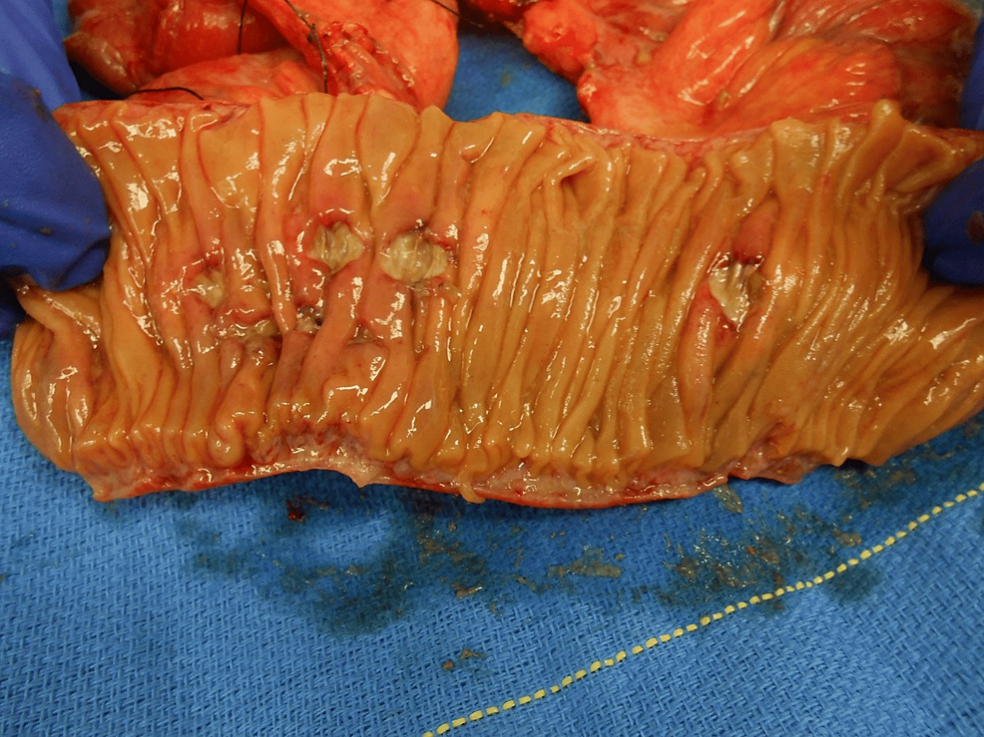
This photograph shows a picture of the resected small bowel demonstrating multiple full-thickness intraluminal ulcerations that formed extraluminal fibrinous exudates.

**Figure 4:**
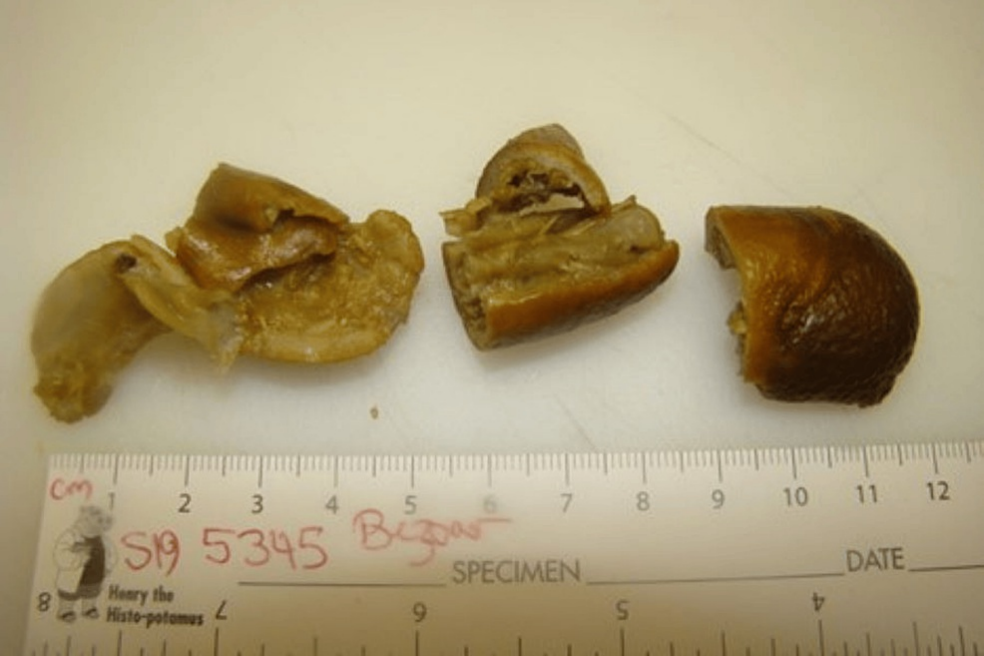
This photograph shows components of the ingested kumquats retrieved from the terminal ileum.

The patient was emergently taken to the operating room for an exploratory laparotomy. Multifocal areas of the small bowel with fibrinous exudate and full-thickness ulcerations were encountered along the distal jejunum and proximal ileum (Figure 3). The terminal ileum contained a partially obstructing soft intraluminal mass measuring approximately 8 × 3 cm. A total of 70 cm of non-viable small bowel was excised along with the intraluminal mass, which had been gently milked into the proximal ileum and demonstrated that it was in several pieces (Figure 4). This resected bowel included a portion of the distal jejunum and proximal ileum. Intestinal continuity was re-established with a side-to-side stapled anastomosis. Of note, no intra-abdominal adhesions were found, which could have been triggered by the previous radiation therapy. Postoperatively, the patient had an uncomplicated hospital course and was discharged on postoperative day nine. He was evaluated several times on an outpatient basis over the next year and found to have no issues regarding his previous abdominal surgery.

Pathological examination of the resected small bowel demonstrated acute transmural inflammation with scattered ulceration compatible with foreign body obstruction and superimposed bacterial and fungal infection (Figure 3). The soft green-brown-tan intraluminal mass was in multiple pieces and measured in total 6.4 × 2.3 cm. It was found to have high levels of oxalate, which are typical of citrus fruits.

Upon further interview with the patient following the operation about the findings, he admitted to eating whole pieces of unpeeled kumquats from a local market before taking a nap in his car and expected no repercussions from doing this. He awoke with a stomachache and thus drank a small bottle of Maalox®, which helped for a while, but then the pain became severe, prompting his visit to the emergency department at our Veterans Hospital. The total time from this patient's ingestion of kumquats, until he sought medical evaluation, was approximately 24 hours.

## Discussion

We report a highly unusual case of small bowel obstruction from ingestion of a citrus fruit, known as kumquats, which led to intestinal perforation and peritonitis. Since the fruit was unpeeled and not easily digestible, it acted like a bezoar, which is a solidified collection of inedible or undigested material in the gastrointestinal tract, having a reported incidence of approximately 1% or less in the general population [5]. Several types of bezoars exist based on their composition, but phytobezoars (composed of indigestible food particles that are found in vegetable or fruit fiber) are the most common. Other types of bezoars include trichobezoar (hair), phamacobezoar (various medications or pills), lactobezoar (milk or milk products), and other indigestible food materials [6]. Although approximately 78% of bezoars are identified in the stomach, as many as 17% or more can be found in various portions of the intestine, most commonly the jejunum and ileum [6].

Management of obstructive bezoars is dependent on the type of bezoar and its location. Therapies are categorized as dissolution, fragmentation, or retrieval. Bezoars in the proximal gut (often phytobezoars) are frequently amendable to dissolution or endoscopic removal. Not uncommonly, Coca-Cola® administration has been found effective for dissolution [6,7]. However, distal bezoars usually require surgical intervention for retrieval. Rapid treatment is necessary for a phytobezoar since possible complications include ulceration, bleeding, bowel obstruction, and perforation [7].

**Figure 5:**
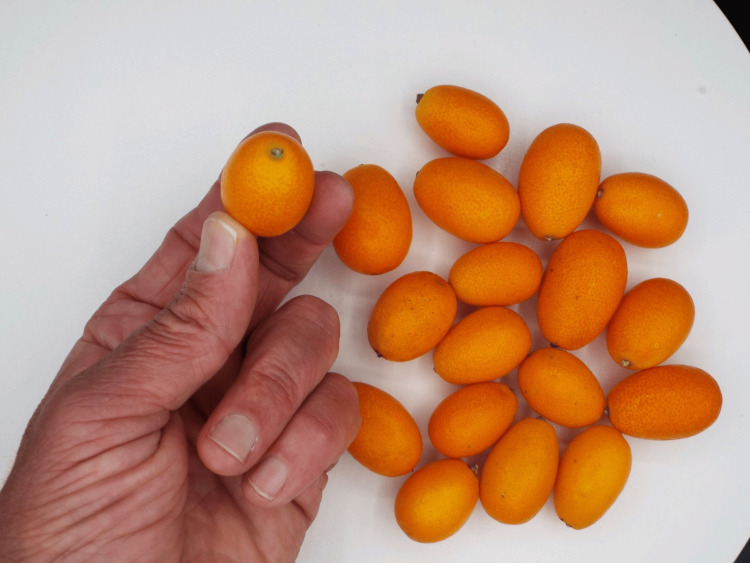
How the kumquat fruit typically looks in comparison to the human hand (photograph courtesy of Gregory Labenz and Dr. Thomas Miller, 2024).

In our patient, kumquat ingestion was responsible for his clinical presentation. The kumquat fruit belongs to the family of citrus fruits and is similar to the orange in color and texture but is considerably smaller, varying in size from 2.5 to 5 cm (1-2 inches) in diameter (see Figure 5) [8]. On average, one serving (four to five kumquats) possesses about 70 calories and is an excellent source of vitamin C as well as approximately 6.5 g of dietary fiber. It is often eaten whole. It elicits a taste that is predominately sour with a sweet aftertaste when chewing the skin along with the fruit. Because of the thickness of the pulpy skin, it should be chewed carefully when eaten whole, and as with all high-fiber foods, accompanied with plenty of water so that when swallowed, it is easily digested. Unfortunately, our patient failed to do that.

Precisely how our patient's kumquat ingestion contributed to all aspects of the clinical findings remains uncertain. It is quite clear that undigested pieces of kumquats got stuck in the terminal ileum (see Figure 4) and thereby induced a bowel obstruction. But how did this citrus fruit induce mucosal ulcerations, transmural inflammation, and perforations in the distal jejunum and proximal ileum? Since the patient himself stated that he swallowed the kumquats nearly whole or, at best, only partially eaten, it is our contention that they ultimately accumulated in sections of the small intestine with narrowed lumens (such as the distal jejunum and proximal ileum) acting as a bezoar and blocking the passage of digestive fluids. In time, these citrus peels, which contain citric acid and oxalates, irritate the delicate lining of the intestinal mucosa, triggering an inflammatory response. In addition, the bezoar effect of these kumquats also caused pressure necrosis of the bowel wall by compromising blood flow. The resultant lack of oxygen supply and essential nutrients elicited tissue inflammation, eventually causing perforations. Resident bacteria in the lumen could then lead to bacterial overgrowth and release of toxins that would further irritate the intestinal wall and worsen the inflammation. Ultimately, the kumquat peels would work their way distally to reside in the terminal ileum where they were found at operation. Obviously, this sequence of events cannot be proved beyond doubt, but it is consistent with the clinical course of our patient and points out the serious consequences that a bezoar can elicit.

## Conclusions

Foreign body small bowel obstructions are rare, and a high level of clinical suspicion is necessary when patients do not have a predisposing factor for obstructive symptoms. In our patient, we were totally surprised with the findings until he ultimately admitted to his ingestion of unpeeled kumquats. While this citrus fruit is very tasty and an excellent source of vitamin C and fiber, it must be remembered that it possesses a thick, pulpy skin that does not digest very easily. As such, we recommend that it should be peeled first before enjoying the fruit. Hence, the axiom, “Avoid the Peel!” For those who insist on eating it whole, it must be chewed carefully before swallowing and accompanied by plenty of water so that it is easily digested and does not act as a bezoar as it did in our patient.
